# Human Metapneumovirus Attachment Protein Contributes to Neutrophil Recruitment into the Airways of Infected Mice

**DOI:** 10.3390/v9100310

**Published:** 2017-10-22

**Authors:** Nagarjuna R. Cheemarla, Antonieta Guerrero-Plata

**Affiliations:** 1 Department of Pathobiological Sciences, Louisiana State University, Baton Rouge, LA 70803, USA; ncheem1@lsu.edu; 2 Center for Experimental Infectious Disease Research; Louisiana State University, Baton Rouge, LA 70803, USA

**Keywords:** human metapneumovirus, HMPV, lung, paramyxovirus, neutrophils, attachment protein, G protein, respiratory, mouse

## Abstract

Human Metapneumovirus (HMPV) is a leading respiratory pathogen that causes lower respiratory tract infections worldwide. Acute HMPV infection induces an exacerbated inflammatory neutrophilic response leading to bronchiolitis and pneumonia. However, the mechanism by which the virus regulates neutrophil infiltration into the airways still remains unexplored. In this work, we used an experimental mouse model of HMPV infection to demonstrate that the attachment (G) protein of HMPV contributes to the recruitment of neutrophils into the airways and modulate the production of neutrophil chemoattractants and Type I IFN responses, specifically IFN-α. These findings provide the first evidence that the HMPV G protein contributes to the in vivo neutrophilic response to HMPV infection and furthers our understanding on virus induced inflammatory responses in the airways.

## 1. Introduction

Human metapneumovirus (HMPV) is a single negative-stranded RNA, enveloped virus classified in the *Paramyxoviridae* family. Its genome, of 13,335 nt, codes for nine different proteins: the fusion (F), attachment (G), small hydrophobic (SH), nucleocapsid (N), phosphoprotein (P), polymerase (L), matrix (M), and second matrix (M2-1, M2-2) [1]. HMPV was first identified in 2001 from nasopharyngeal aspirates of hospitalized infants [2], and has soon emerged to be a leading respiratory pathogen worldwide infecting infants, elders, and immunocompromized individuals [3]. Epidemiological data indicate that this respiratory virus represents a major respiratory pathogen worldwide. HMPV is responsible for 5 to 15% of pediatric hospitalizations for respiratory tract infections [4,5,6,7]. Indeed, it is second only to Respiratory Syncytial virus (RSV) infection in infants admitted with lower respiratory tract viral infections causing mortality and morbidity [4,8,9,10]. In elderly adults aged ≥65 years old, HMPV accounts for about 4.1% hospitalizations with respiratory tract infections, impacting more severely those subjects with underlying conditions, such as cardiovascular diseases, organ transplantation, or other hematologic malignancies [11,12,13,14]. One hallmark of HMPV infection is that it is characterized by aggravated inflammatory responses leading to bronchiolitis and pneumonia [8]. Currently there is no approved vaccine available to protect from HMPV infection.

Inflammatory outcomes during HMPV infection are mediated by virus-induced cytopathology and the secretion of cytokines and chemokines [15,16]. Clinical evidence indicates that HMPV induces neutrophil infiltration and associated mediators within the airways of infants with bronchiolitis [17], providing evidence of the neutrophilic inflammatory response in vivo and highlighting the importance of these cells as a potential target of therapeutic intervention for treatment of bronchiolitis in infected children. The increased neutrophil infiltration by HMPV in the airways has been reproduced in the mouse model of infection, including in adult [18,19,20] and aged mice [21], where HMPV infection induces similar neutrophil recruitment into the airways of both age groups of mice [21]. However, the role of HMPV in regulating the recruitment of neutrophils to the lungs remains elusive. On the other hand, the interferon (IFN) response appears to regulate the neutrophil infiltration in some viral [22,23] and bacterial [24] infections, as well as in tumor bearing mice [25,26]. In that regard, HMPV infection induces a robust production of type I interferon in infected mice [27,28], which appears to be regulated by the expression of the HMPV attachment protein (G protein) [28,29]. Therefore, we reasoned that HMPV G protein contributes to the neutrophil recruitment into the airways during HMPV infection through the IFN response. For that, we used an experimental mouse model to quantify IFN-α production, neutrophil recruitment, and chemokine response to a recombinant HMPV lacking the G protein. We found that the lack of the attachment protein increased the production of IFN-α but decreased the production of neutrophil chemoattractants and the recruitment of neutrophils to the alveolar spaces. These findings suggest a key role for HMPV attachment (G) protein in contributing to the inflammatory responses in vivo.

## 2. Materials and Methods

### 2.1. Virus Stocks

Recombinant HMPV lacking the attachment G protein (rHMPV-ΔG) and full-length recombinant HMPV (rHMPV) were generated by reverse genetics, as we previously described [28]. The viruses were grown and titrated in LLC-MK2 cells (ATCC, Manassas, VA, USA) in the presence of trypsin (Worthington, Lakewood, NJ, USA). Viruses were sucrose purified and not used beyond passage 5. [28]. In some experiments, rHMPV was exposed for 10 min to UV irradiation, as previously reported [30].

### 2.2. Ethic Statement

Animal care and use were conducted in accordance with the National Institutes of Health and Louisiana State University institutional guidelines. The Louisiana State University Animal Care and Use Committee specifically approved this study under the protocol number: 15-062 (15 October 2015). Mice were housed in a temperature-controlled room with proper darkness-light cycles, fed with a regular diet, and maintained under the care of the Division of Laboratory Animal Medicine facility, Louisiana State University, Baton Rouge, LA. The mice were sacrificed by an intraperitoneal injection of ketamine and xylazine, and exsanguinated via the femoral vessels.

### 2.3. Mice and Infection Protocol

BALB/c mice were purchased from Harlan Laboratories. Female 8- to 12-week-old mice were used in all of the experiments. Mice were anesthetized with a combination of ketamine and xylazine, and infected intranasally with 50 μL of hMPV diluted in phosphate-buffered saline. A final administration dose of 5 × 10^4^ PFU/mouse was used for the recombinant virus infections. Mock-infected mice received 50 μL total volume of PBS. 

### 2.4. Mouse Sample Collection

Mice were euthanized by intraperitoneal injection of ketamine and xylazine, and exsanguinated via the femoral vessels, as previously described [20,28]. Bronchalveolar lavage (BAL) samples were collected by flushing the lungs twice with 1 mL PBS and centrifuged 3500 rpm for 5 min at 4 °C. Cell-free BAL supernatants were stored at −75 °C until further analysis. For viral gene expression using qRT-PCR, lung tissue was snap frozen in liquid nitrogen and stored at −75 °C until further analysis.

### 2.5. Differential Leukocyte Counts

Bronchalveolar lavage (BAL) fluid was used for differential leukocyte counts using cytospin methods. Cytospin samples were subsequently prepared from BAL cells and dyed with Wright-Giemsa staining and subjected to differential leucocyte counts under a light microscope. A total of 200 cells per slide were counted. The total cell numbers were enumerated from BAL cell counts obtained by trypan blue exclusion.

### 2.6. Detection of Cytokines and Chemokines

Levels of cytokines and chemokines in BAL fluid were determined with the Milliplex MAP^TM^ 32-Mouse-Plex cytokine detection system (Millipore, Billerica, MA, USA), according to the manufacturer’s instructions. The panel included the following cytokines: IL-1α, IL-1β, IL-2, IL-3, IL-4, IL-5, IL-6, IL-7, IL-9, IL 10, IL-12 p40, IL-12 p70, IL-13, IL-15, IL-17, M-CSF, G-CSF, GM-CSF, IFN-γ, TNF, CXCL1, CXCL2, CXCL5, CXCL9, CXCL10, CCL2, CCL3, CCL4, CCL5, CCL11, LIF, and VEGF. The range of sensitivity of this assay is 3.2 to 10,000 pg/mL. Cell-free BAL supernatants were also tested for the production of type I (IFN-α) interferon using ELISA tests according to the manufacturer’s instructions (PBL Assay Science, Piscataway, NJ, USA).

### 2.7. Real-Time qRT-PCR

Lung tissue stored at −75 °C was used for viral gene expression using qRT-PCR, as described previously [13]. Briefly, RNA was extracted from the lung tissue using RNeasy Plus kit (Qiagen, Hilden, Genmany) and viral gene expression was determined using specific primers and probes (Integrated DNA Technologies, Coralville, IA, USA) on a 7900HT Fast Real-Time PCR following manufacturer’s instructions. Expression of target genes was quantified using the comparative cycle threshold method and results were normalized to the endogenous GAPDH with expression levels normalized to transcripts from mock-infected mice.

### 2.8. Statistical Analysis

Statistical significance was calculated by unpaired t test and one-way ANOVA to ascertain the differences between the animal groups, followed by a Tukey-Kramer test to correct for multiple comparisons using Graph Pad InStat 3 (GraphPad Software, La Jolla, CA, USA).

## 3. Results

### 3.1. Inhibition of IFN-α Responses by HMPV G Protein In Vivo

**Figure 1:**
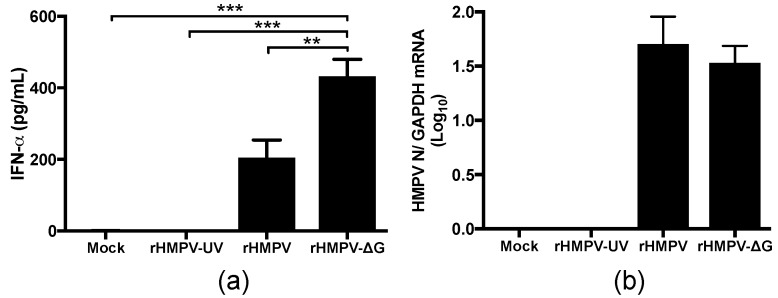
Human Metapneumovirus (HMPV) G protein regulates interferon responses in vivo. (**a**) BALB/c mice were infected with rHMPV-ΔG or rHMPV and Bronchalveolar lavage (BAL) collected at day 1 after infection. Interferon-α (IFN-α) responses were determined by ELISA in BAL collected at day 1 after infection; (**b**) HMPV viral gene expression in infected mice. BALB/c mice were infected with rHMPV-ΔG or rHMPV and lung tissue collected at day 1 after infection. Expression of HMPV N was done by qRT-PCR. *n* = 4–10 mice/group. Mean ± SEM are shown. ** *p* < 0.01, *** *p* < 0.005.

Previous studies in vitro have identified HMPV G protein as a negative regulator of the IFN response [28,29]. In order to determine the effect of the HMPV G protein in the IFN response in vivo, BALB/c mice were infected with rHMPV, rHMPV-∆G or mock infected. After 24 h of infection, BAL supernatants were collected and tested for the IFN production by ELISA. As shown in Figure 1a, the lack of the attachment glycoprotein resulted in a significant increase in the production of IFN-α. We observed that mice infected with rHMPV-ΔG induced a 1.3-fold increase in the production of IFN-α as compared to full-length rHMPV infected mice. However, no difference in viral gene expression was noted when compared between rHMPV and rHMPV-∆G after 24 h of infection (Figure 1b), confirming that the infection of mice with rHMPV and rHMPV-∆G was comparable. Thus, validating the inhibitory effect of G protein on the IFN response. Furthermore, in order to confirm that the observed production of IFN-α by rHMPV was due to the viral infection, a group of mice were inoculated with UV-inactivated rHMPV and the production of IFN was determined. Our data show that the inoculation of mice with UV-inactivated rHMPV failed to induce any production of IFN-α showing that IFN-α production was dependent on the rHMPV replication (Figure 1a). Together, these results demonstrate that HMPV-G protein regulates IFN-α responses in vivo.

### 3.2. HMPV G Protein Contributes to Neutrophil Recruitment

**Figure 2:**
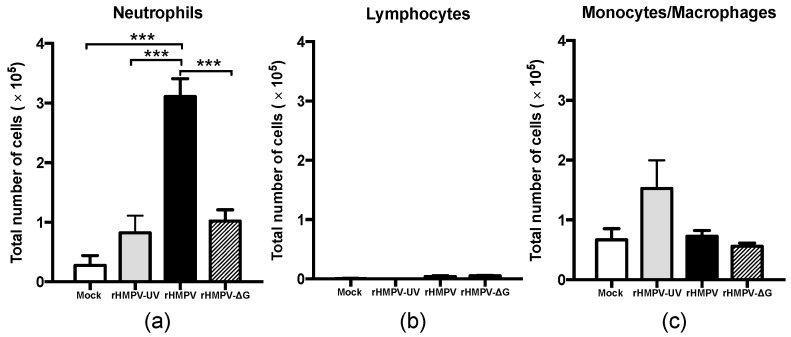
HMPV G protein contributes to neutrophil infiltration into the airways of infected mice. BALB/c mice were infected with rHMPV-ΔG or rHMPV and BAL collected at day 1 after infection. Total number of (**a**) neutrophils; (**b**) lymphocytes and (**c**) monocytes/macrophages was determined by cytospin analysis and total number of cells enumerated by total BAL cell counts. *n* = 4–10 mice/group. Mean ± SEM are shown. *** *p* < 0.005.

To determine whether the HMPV attachment glycoprotein plays a role in the recruitment of neutrophils, BAL samples were collected 24 h after inoculating mice with rHMPV, rHMPV-∆G or PBS, and differential cell analysis was performed. We focused on the neutrophil recruitment at 24 h based on previous observations that indicate that neutrophil recruitment peaks at day 1 after HMPV infection [20]. As shown in Figure 2a, analyses of cytospin preparations revealed a significant decrease in the total number of neutrophils recruited to alveolar spaces in mice infected with rHMPV-ΔG (1.0 × 10^5^ ± 0.2) when compared with those with rHMPV infection (3.1 × 10^5^ ± 0.3), indicating that G protein contributes to the recruitment of neutrophils. Also, the levels of recruitment of neutrophils in rHMPV-ΔG (1.0 × 10^5^ ± 0.2) were comparable to mice infected with UV light inactivated rHMPV (0.8 × 10^5^ ± 0.3). On the other hand, there was no evident change in the recruitment of monocytes/macrophages (Figure 2c) or lymphocyte population (Figure 2b), suggesting that G protein contributes mainly to the recruitment of neutrophils to the alveolar spaces.

### 3.3. HMPV G Regulates Lung Cytokine and Chemokine Profile in the Lungs of Infected Mice

**Figure 3:**
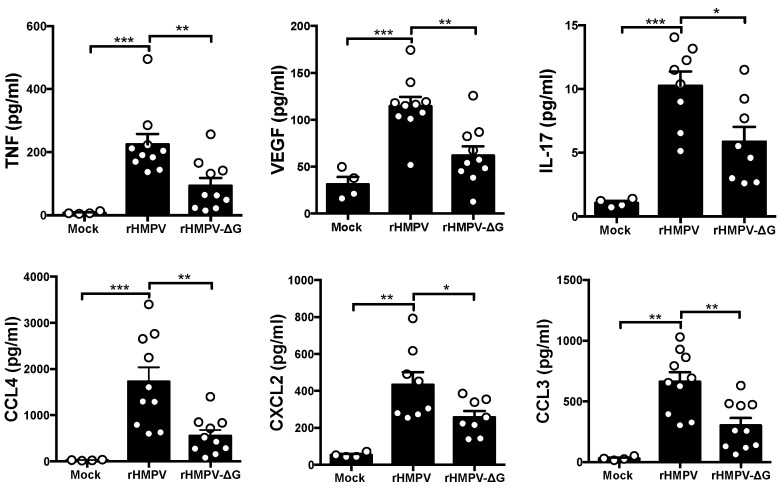
HMPV G alters cytokine profile in infected mice. BALB/c mice were infected with rHMPV-ΔG or rHMPV and BAL collected next day. BAL samples from each group of mice were assessed for cytokine/chemokine production by a multi-Plex cytokine detection system. *n* = 4–10 mice/group. Mean ± SEM are shown. * *p* < 0.05, ** *p* < 0.01, *** *p* < 0.005.

To further elucidate the role of G protein in the production of neutrophil chemoattractants and proinflammatory cytokines, we sought to assess the production of lung cytokine and chemokine profile after 24 h of rHMPV-ΔG infection and compare it to that of rHMPV. Cell-free supernatants from BAL samples were analyzed by multiplex assay, using multi-Plex cytokine detection, as described in methods. As shown in Figure 3, the levels of TNF (↓58%), IL-17(↓44%), CXCL2(↓40%), VEGF (↓46%), CCL3(↓54%), and CCL4 (↓68%), were decreased (as indicated) in mice infected with rHMPV-ΔG as compared with those infected with full-length rHMPV, suggesting that G protein contributes to the production of these chemokines in vivo. However, when compared between both groups of infected animals, no significant difference was observed in the production of IL-1α, IL-1β, IL-4, IL-5, IL-6, IL-9, IL 10, IL-12 p70, IL-13, IL-15, M-CSF, G-CSF, GM-CSF, IFN-γ, CXCL1, CXCL5, CXCL9, CXCL10, CCL2, CCL5, CCL11, and LIF. Other cytokines were not induced by the recombinant viruses in the infected mice (IL-2, IL-3, IL-7, and IL-12 p40).

## 4. Discussion

The regulation of the inflammatory immune response by infectious agents involves several factors from the host and the pathogen. Viral infections are known to alter the innate immune response [31,32]. In fact, several viral proteins are known to inhibit the IFN response in vitro [29,33,34] and in vivo [35]. In that regard, despite previous studies have demonstrated that the attachment glycoprotein (G) of HMPV inhibits the IFN response in vitro [28,29], this work demonstrates, for the first time, that the HMPV G protein inhibits the IFN response in vivo. Due to the restriction of high viral titers of the purified recombinant viruses, the final administered viral inoculum was 5 × 10^4^ PFU/mouse. However, that amount of viral inoculum was enough to induce an IFN-α response that allowed us to define the inhibitory effect of the HMPV G protein in vivo. That inhibitory effect was validated by the fact that both viral inoculums (rHMPV and rHMPV-∆G) were comparable as they were purified and titrated by the same methods, and no difference was observed in the viral gene expression when measured in the lung samples from the infected mice (Figure 1b).

There is evidence that interferons have pleiotropic immune functions in several models. In an experimental mouse model, type I IFN suppresses neutrophil recruitment by negatively regulating CXC chemokine expression in influenza [23,36], herpes simplex-1 [22], *Listeria monocytogenes* [24], and tumor-associated diseases [26]. Thus, the observed increased production of IFN-α by rHMPV-ΔG, due to its regulatory effect [22,37], may contribute to the suppression of the recruitment of neutrophils induced by HMPV. On this subject, several studies indicate that neutrophils are the predominant cell population recruited to the alveolar spaces in HMPV-infected mice during the early phase of the infection (Figure 2 and [18,19,20]). However, to the best of our knowledge, these findings represent the first evidence that the HMPV attachment protein contributes to the recruitment of neutrophils into the lungs. Moreover, the UV treatment of rHMPV significantly reduced the number of neutrophils, indicating that the recruitment of these cells is dependent on viral replication and the de novo synthesis of the G protein. These observations are in line with studies with other respiratory viruses, including those with respiratory syncytial virus (RSV), an HMPV-close-related human paramyxovirus, in which it has been shown that the fusion (F) protein, of the RSV 2–20 strain, contributes to the infiltration of neutrophils into the lungs of infected mice [38]. Similarly, influenza A virus, a ssRNA ortomyxovirus, appears to regulate neutrophil infiltration into the alveolar spaces through the expression of the PB1-F2 protein [39]. This suggests that different surface viral proteins can specifically contribute to the infiltration of neutrophils to the respiratory tract. 

The observed contribution of the HMPV G protein to the recruitment of neutrophils in the infected mice, suggest that G protein may also regulate the expression of those cytokines and/or chemokines that control the recruitment of neutrophils to the alveolar spaces. Opposite to the effect of HMPV G protein on the IFN response, we observed some changes in CCL3, CCL4, VEGF, TNF, IL17, and CXCL2, which are all recognized mediators involved in the neutrophil recruitment to the sites of insult. In this work, we observed that the lack of G protein in HMPV resulted in the reduced expression of TNF and IL-17, which are known to promote the expression of neutrophil chemotactic cytokines. In fact, IL-17 together with TNF can synergistically induce the endothelial expression of neutrophilic chemokines including CXCL2 [40], which may explain also the reduced expression of CXCL2 observed in the rHMPV-ΔG-infected mice. In addition, we speculate that due to its suppressive effect on TNF [41], the IFN-α response in rHMPV-ΔG could be linked to the reduced production of TNF. In the same context, IFN-α has been reported to inhibit IL-17 production in PBMC’s from patients with chronic active Hepatitis B infection (CAHB), suggesting the pleiotropic effect IFN-α has on proinflammatory cytokines associated with neutrophil activation and chemotaxis [42]. Moreover, type I IFN has also been shown to repress CXCL2 production in in vivo [23] and in vitro [36] settings, suggesting that the increased IFN-α response in rHMPV-ΔG could contribute to the observed diminished production of CXCL2. Furthermore, the absence of G protein led to a decrease in the expression of VEGF, which could also impact the neutrophil numbers to the site of infection since VEGF contributes to the recruitment of proangiogenic neutrophils from the circulation to the tissues [43]. These results are in line with data from melanoma studies in vitro, where IFN-α treatment significantly reduced the expression of VEGF suggesting a suppressive effect of Type I IFN on this cytokine [44,45]. Finally, the neutrophil recruitment after rHMPV-ΔG infection could have also been altered by the reduced expression of CCL3 and CCL4, which are neutrophil-active chemokines [46]. However, studies in vitro in an epithelial cell line (A549) indicate that the HMPV G protein rather inhibited the expression of CCL3 and some other cytokines [29]. This discrepancy might be due not only to the inherent differences of the experimental models, but also to the exerted effect of HMPV G protein on the complex microenvironment in vivo, where several cellular populations, including macrophages, lymphocytes, endothelial, and epithelial cells, mediate the overall cytokine/chemokine production. In fact, similar observations to the current work have been reported in a mouse model of pneumonia virus, where the nonstructural (NS) proteins NS1 and NS2 (rPVM ∆NS1∆NS2; rPVM ∆NS2) antagonize IFN responses in vivo, but on the other hand, induced lower amounts of proinflammatory cytokines in the airways when compared to rPVM virus [35]. However, data on viral proteins regulating neutrophilic responses to the sites of inflammation is limited and further work is warranted. Overall, data from this work suggest that the attachment protein of HMPV regulates neutrophil recruitment to the lungs by modulating the production of neutrophil chemoattractants.

In summary, the above results demonstrate a novel role for the attachment protein of HMPV as a contributing factor for neutrophil recruitment to the sites of infection. This effect appears to be influenced by the regulation of neutrophil chemoattractants and an exacerbated response of type I IFN production. We have recently demonstrated that neutrophils exert a protective effect to HMPV-induced pathogenesis in mice [20]. However, an exacerbated accumulation of neutrophils contribute to severe pulmonary inflammation [17]. Therefore, a controlled balance of neutrophil accumulation in the airways after HMPV infection would be helpful for the outcome of the infected individuals. In this regard, the attachment protein of HMPV represents an attractive target for future therapeutic applications to reduce an excessive accumulation of neutrophils in the airways.
